# An Unresolving Case of Pyomyositis: A Case Report

**DOI:** 10.5704/MOJ.2307.011

**Published:** 2023-07

**Authors:** G Dhivakaran

**Affiliations:** Department of Orthopaedics and Traumatology, Hospital Duchess of Kent, Sandakan, Malaysia

**Keywords:** pyomyositis, skeletal muscle, immunosuppressed, rare, pulmonary tuberculosis

## Abstract

Pyomyositis which is also known as myositis tropicans is a rare condition where there is bacterial infection of the skeletal muscle. Its manifestation includes pain and tenderness of the affected muscle and general infective symptoms. It commonly occurs in immunocompromised individuals and patients with previous history of trauma to the affected muscle. We report a case of a 16-year-old boy with history of underlying bronchial asthma who presented with multiple abscesses. He underwent multiple operations to drain the infection and targeted antibiotic therapy subsequently. Despite undergoing surgical debridement, drainage and antibiotic treatment, he was still having repeated bouts of fever and his inflammatory markers were not reducing. He was then diagnosed with concurrent pulmonary tuberculosis infection which subjected him to an immunosuppressed state thus arising to the condition of pyomyositis and unresolving fever. The patient then made prompt improvement when the underlying cause of immunosuppression; pulmonary tuberculosis was treated as well.

## Introduction

Pyomyositis is a rare infection of the skeletal muscle that often coincides with abscess formation deep within large striated muscles^1^. It commonly affects large muscle groups in the lower limb. It is believed to occur more commonly in immunocompromised individuals although it can be caused by factors affecting the muscle itself such as strenuous exercise and direct trauma to the muscle. The bacteria invade skeletal muscle during bacteraemia, dental manipulation or even contaminated lower urinary tract infection. The most common causative organism is *Staphylococcus aureus* accounting for 70% of cases in tropical countries^2^. Group A streptococcus accounts for another 1% - 5% of cases. Most cases have been reported in tropical countries however the incidence of this disease in temperate climates are on the rise. It is believed to be due to increased awareness of the disease, increase in the population of immunocompromised patients and improvement of diagnostic methods. The infection is believed to spread hematogeneously and can be rapidly progressive from a localised infection to severe sepsis in a matter of days. Diagnosis is a challenge as it often presents with non-specific symptoms such as vague musculoskeletal pain and due to a wide array of differential diagnosis. Recent literature reports that the time of diagnosis from admission is around 7-10 days because the affected muscles are deeply situated, and local signs are rare.

The mainstay of treatment of pyomyositis is surgical drainage of the abscess and targeted antibiotic therapy. Intravenous antibiotics are usually given for three to four weeks but the duration can vary based on the clinical condition of the patient.

## Case Report

**Fig 1:**
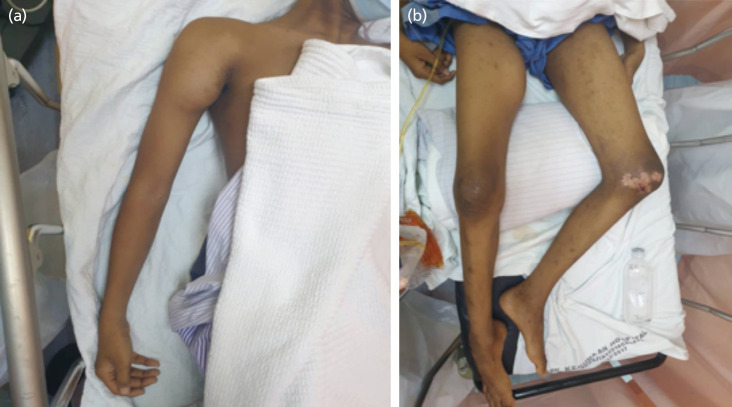
(a) Large erythematous swelling involving his right shoulder. (b) Tender fluctuant swelling involving medial aspect of right thigh and left anterior thigh.

We report the case of a 16-year-old boy with an underlying history of childhood bronchial asthma not on proper follow-up or any regular steroid and no prior history of hospitalisation that complained of left knee swelling for two weeks. The swelling was initially firm, tender and erythematous that subsequently progressed to have a punctum with pus discharge. His range of motion was limited due to pain however he was still able to ambulate without aid. He denied any history of fever, trauma or fall. He visited a nearby private clinic and was given a one-week course of oral antibiotics and analgesia. He presented to the emergency department a week after that due to worsening of symptoms. He started having erythematous painful swelling at the inner aspect of his right thigh and right shoulder (Fig. 1). He also started having fever for six days associated with a two weeks history of poor oral intake and generally feeling lethargic. Additional relevant information includes no history of malignancy or diabetes in his family. He denied any high-risk behaviour, usage of illicit drugs or any constitutional symptoms.

His initial assessment upon presentation showed that the patient was septic. He was febrile with a blood pressure of 91/57mmHg and tachycardic with a pulse rate of 138bpm. He was resuscitated accordingly with intravenous fluids. Upon further examination, he had multiple abscesses throughout his body. He had a 3cm x 5cm tender and fluctuant swelling at the proximal aspect of right arm extending to the anterior aspect of right shoulder joint (Fig. 1). Range of motion of the right shoulder joint was reduced due to pain. He also had a 10cm x 15cm tender and fluctuant swelling at the medial aspect of right thigh extending to posterior aspect of the thigh and gluteal region (Fig. 1). The range of motion of the right hip joint was reduced due to pain as well. His left lower limb had a 5cm x 5cm tender and fluctuant swelling of the anterior aspect of left thigh and knee. Relevant laboratory investigations were sent and he was given a dose of broad-spectrum antibiotic by the emergency department; IV Amoxicillin/Clavulanic acid.

**Fig 2:**
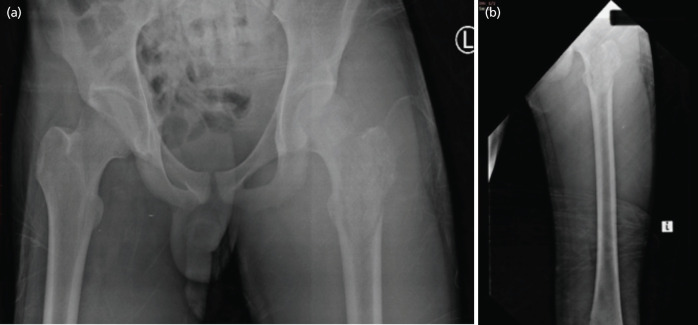
(a) AP view of pelvis and (b) lateral radiograph of left femur showing sclerosis of the greater trochanter of left femur.

Laboratory investigations showed a markedly elevated white blood cell count at 22.8 x 10^3^/uL with neutrophil predominance. His erythrocyte sedimentation rate was >120mm/H and C-reactive protein was elevated as well at 256mg/L. He was also having normocytic normochromic anaemia with a haemoglobin of 8.3g/dL. Peripheral blood film attributed the anaemia to chronic illness. He had coagulopathy attributed to sepsis with an INR of 1.32. A blood culture, melioidosis serology and infective screen were taken prior to commencement of antibiotics. Plain radiographs were ordered of the particular affected limbs, and it showed osteomyelitis changes of the proximal left femur (Fig. 2). We proceeded with an ultrasound which showed multiple intramuscular collections; Right shoulder measuring 3.5cm x 5.6cm x 14.4cm. Medial compartment of right thigh measuring 5.8cm x 5.9cm x 14.4cm. Right lateral gluteal region measuring 1.5cm x 4.6cm x 5.1cm. Left lateral gluteal region extending to distal thigh measuring 3.3cm x 8.0cm x 22.3cm. Left inner thigh measuring 3.9cm x 5.9cm x 7.1cm. Subcutaneous collection seen anterior to tibial tuberosity and distal patellar tendon measuring 0.7cm x 1.7cm x 2.8cm.

In the emergency department, his anti-infective was changed to Ceftazidime to cover for melioidosis as it is an abscess producing organism that is endemic in this part of Malaysia. After reviewing his ultrasound report, he was then pushed to the operation theatre for incision and drainage and left femoral canal washout for multiple abscesses and osteomyelitis of the proximal femur. Intra-operatively, we drained 200cc of pus from subcutaneous region of right shoulder that was not communicating to the joint. A 200cc of pus from subcutaneous region of medial right thigh. 500cc pus from intra-muscular region of posterior right thigh that was not extending beyond inguinal region. A 1.2L of pus from lateral aspect of left hip. The musculature at the affected levels were unhealthy with poor colour but still had good contractility. The unhealthy fascia at the affected layers were debrided as well. The greater trochanter of the femur was unhealthy with an area of necrosis. There was also poor colour and contractility of the gluteus medius muscle that was detached from the greater trochanter. We debrided the greater trochanter until there was punctate bleeding seen and washed out the femoral canal as well with copious amount of normal saline. The muscles and fascia at this level were debrided accordingly as well. We also drained 10cc subcutaneous pus drained from anterior thigh and 5cc of superficial pus from infrapatellar region. Samples of pus, tissue and muscle for gram stain, culture and sensitivity and acid-fast bacilli were sent. All wounds were packed with povidone saline. He was then sent to the ward. Intravenous Ceftazidime was continued.

His blood culture, pus, bone, tissue, intramedullary tissue and muscle tissue from medial aspect of right thigh grew Staphylococcus aureus. This was when we established a diagnosis of pyomyositis based on growth from his muscle tissue. His acid-fast bacilli stain from pus, bone and tissue to look for tuberculous organism showed no acid-fast bacilli. Antibiotics were changed to Cloxacillin as per the intra-operative antibiotic sensitivity report. His operative wounds were dressed daily with normal saline and paraffin gauze in ward. In view of the patient having MSSA bacteraemia, we proceeded with an ultrasound of his abdomen and echocardiogram of his heart. There was no evidence of intra-abdominal collection or vegetations in his heart.

The patients general condition improved in the subsequent days. The range of motion of the shoulder joint, left hip and knee joint were approaching near normal. His inflammatory markers were repeated on day four post-operation. His white cell count reduced from 22.8 x 10^3^/uL during admission to 16.37 x 10^3^/uL. His erythrocyte sedimentation rate reduced from >120mm/H to 91mm/H and C-reactive protein from 256mg/L to 80mg/L. Intravenous Cloxacillin was continued with daily dressing of the surgical wounds.

However, on day six post-operation, he started having tender and fluctuant swelling at his right deltoid region; lateral to his initial abscess, anterior aspect of left arm and left leg. It was highly suggestive of an abscess clinically and he was having low grade temperature with rising white blood cell count from 15.39 x 10^3^/uL → 16.46 x 10^3^/uL → 21.87 x 103/uL. He was then sent to the operation theatre again for incision and drainage of the newly developed abscesses at the right shoulder, left arm and left leg and wound debridement and delayed primary closure of the right shoulder and right thigh wound from the first operation. Since the left hip wound from the first operation was clean, it was decided for vacuum assisted closure application of the left hip wound. Intra-operatively, we drained 8cc intramuscular pus discharge from left arm, 5cc subcutaneous pus discharge from left leg, 3cc subcutaneous pus discharge from anteromedial aspect of left thigh, 7cc subcutaneous pus from right shoulder.

After his second operation, his wounds were dressed daily. There was no evidence of infection from his wound clinically and he was put on total of two cycles of vacuum assisted closure dressing for his left hip wound. However, after completing two weeks of Cloxacillin, his inflammatory markers were still raised. His C-reactive protein was uptrending from 80mg/L → 115mg/L → 68mg/L. His erythrocyte sedimentation rate trend was 91mm/H → 114mm/H. All cultures from the second operation grew mixed growth of organisms except intramedullary tissue which grew Pseudomonas aeruginosa. Intravenous antibiotics were then changed to Piperacillin/tazobactam based on the clinical condition of the patient and the latest culture and sensitivity report. The patient’s condition improved subsequently. He was able to ambulate in the ward without aid. His WBC trend was 13.87 x 10^3^/uL → 6.78 x 10^3^/uL. Two repeated blood cultures taken after the initial one that grew Staphylococcus aureus did not grow any organism. He was then sent to the operation theatre for delayed primary closure of all his surgical wounds. This was 15 days after his second operation.

After his third operation despite his favourable wound conditions, the patient developed multiple episodes of temperature spikes in the ward. We were thinking in the lines of him developing a hospital acquired infection. A full septic workup was repeated. Urine culture and blood culture showed no growth. Erythrocyte sedimentation trend was 98mm/H → 88mm/H → 85mm/H. C-reactive protein trend was from 31mg/L → 64mg/L → 85mg/L. On examination, he was having tender left inguinal lymphadenopathy. We proceeded with an ultrasound that showed reactive lymphadenopathy with no evidence of abscess. His melioidosis, Hepatitis B, Hepatitis C, HIV serology all came back as negative as well. A HbA1c was sent to screen him for diabetes mellitus, and it was normal. We then noticed that his white blood cell count was down going in trend remarkably; 3.81 x 10^3^/uL → 3.38 x 10^3^/uL → 2.45 x 10^3^/uL and he was neutropenic with an absolute neutrophil count trend; 0.77 x 10^3^/uL → 1.39 x 10^3^/uL → 1.10 x 10^3^/uL. Subsequently, we sent a primary immunodeficiency screen for him which was unremarkable and sputum AFB as tuberculosis is endemic in this part of Malaysia. The first sample of sputum AFB came back as positive. We started him on anti-tuberculosis treatment. He did not develop any temperature spikes after initiation of anti-tuberculosis drugs. He was given total antibiotics of six weeks. IV Ceftazidime for four days, IV Cloxacillin for two weeks and IV Piperacillin/tazobactam for three weeks. He was discharged home with culture directed oral Ciprofloxacin for three months for osteomyelitis of the proximal left femur and anti-tuberculosis treatment.

We reviewed him six weeks after discharge. His wounds healed remarkably, and he was back to his premorbid status. His erythrocyte sedimentation rate was 14mm/H. On examination of the left hip joint, the range of motion was full as per his normal hip. There was no reduction in the range of motion of his left hip. However, he had weakness during abduction of the hip with a power grade of four. All other movement of the hip was of full power. Repeated radiograph of the left femur showed no radiographic progression of osteomyelitis. Power and range of motion of his right shoulder joint and right knee joint was also back to normal. We referred him to physiotherapy to strengthen his abductor muscle and reviewed him again in three months. During his next follow-up, he had an improvement of his hip abductor power from four to five. He was able to perform all activity of daily living without aid. His erythrocyte sedimentation rate was 2mm/H and his C-reactive protein was 1mg/L. He was still on maintenance phase of anti-tuberculosis treatment during review.

## Discussion

The epidemiology, clinical presentation and laboratory investigation of the case presented was classical to pyomyositis. The disease is commonly believed to affect tropical countries but is highly under reported in western countries. It accounts for 1% - 4% of hospital admission in tropical countries. Pyomyositis classically presents with intermuscular abscess as seen with our patient, however the hallmark of the disease is actually histopathology sample of myositis from the affected muscle^2^. The blood culture and intra-operative culture of our patient yielded Staphylococcus aureus which is the most common organism implicated in this disease. It is seen in up to 90% of cases in tropical countries and 75% of cases in temperate countries. The pathogenesis of the disease is still unclear. The causative organism is believed to spread in the blood to skeletal muscle tissue during bacteraemia. However, skeletal muscle tissue is believed to be resistant to bacterial infection under normal circumstances because myoglobin sequesters iron which is a requirement for bacteria to proliferate^3^. This would inevitably result in slower growth of the organism allowing the body’s immune system to fend off the infection. Smith and Vickers demonstrated that there were less than 1% of patients with skeletal muscle abscess from a series of 336 patients that were autopsied due to staphylococcal septicaemia which concurs to the fact that abscess formation in skeletal muscle tissue is rare^4^.

There are several factors that increases the likelihood of developing pyomyositis. Trauma to the affected muscle makes it susceptible to hematogenous invasion by bacteria. Literature reports that 20 - 50% of patients report a history of blunt trauma or strenuous exercise of the involved muscle prior to presentation^1^. The patient described above however denied any prior history of trauma. Other risk factor includes individuals with human immunodeficiency virus (HIV) infection, intravenous drug use, diabetes, and malignancy. In regard to the case described above, clinically he did not have any symptoms of malignancy. His blood investigations for HIV and primary immunodeficiency screen was unremarkable as well. He was screened for other virus and pus producing organisms that are endemic in this part of the world such as dengue virus, malaria and melioidosis which came back as negative. However, it was later found out that he was having concurrent pulmonary tuberculosis infection. During active phase of pulmonary tuberculosis, there is transient systemic immunosuppression due to over expression of transforming growth factor beta and interleukin-10^5^. We postulate that this concurrent infection put him in an immunosuppressed state susceptible to developing pyomyositis.

With regards to the case above, he presented in the late stage of pyomyositis which is also known as the septicaemic stage. The clinical picture of pyomyositis is usually divided into three stages which are the invasive stage, suppurative stage and septicaemic stage. Invasive stage is characterised by pain and localised erythema of the affected muscle. Some patients may develop low grade temperature and leukocytosis. They do not usually present at this stage as they usually attribute their symptoms to other conditions such as cellulitis or fibromyalgia. It progresses to the second stage from the second to third week of infection. Abscess forms within the muscle. It is accompanied by high spiking fever and systemic symptoms such as malaise. Laboratory investigations would show a high erythrocyte sedimentation rate, C-reactive protein and white blood cell count. Sometimes, the classical sign of abscess on physical examination may be lacking because the overlying muscle is tense. Regional lymph nodes are not involved at this stage. Approximately 90% of patients presents during this stage of the disease. If the abscess remains untreated, it progresses to the late or septicaemic stage. The infection disseminates leading to septicaemia, septic shock and multiorgan dysfunction. The patient described above presented to the emergency department in the septicaemic stage. His blood pressure was low, he was tachycardic and febrile in the emergency department. He had multiple palpable abscess and lymphadenopathy. His inflammatory parameters were markedly elevated too.

The diagnosis of pyomyositis is difficult as it mimics a wide array of other diseases. Its differential diagnosis includes muscle contusion, septic arthritis, osteomyelitis, cellulitis, muscle hematoma, deep vein thrombosis or malignancy such as osteosarcoma. Therefore, there needs to be a high index of suspicion for any patient that presents with muscle pain and fever for pyomyositis. Early diagnosis and initiation of treatment is essential to improve the prognosis of the disease. Laboratory investigations are non-specific. They show evidence of bacterial infection which may be subjected to many other conditions. In our case, we found similar laboratory investigations as literature reports. Leukocytosis with predominant neutrophil, elevation of acute phase reactants and chronic anaemia. Our patient also had a positive blood culture. It is reported in less than 5% of cases. The gold standard for diagnosis of pyomyositis would be aspiration of pus from the muscle tissue. This was seen intra-operatively in this patient from the right and left thigh abscesses. Furthermore, the cultures in vitro from muscle tissue grew *Staphylococcus aureus* as well which consolidated the diagnosis of pyomyositis. A muscle biopsy should also be taken intra-operatively to exclude other conditions such as osteosarcoma.

Plain radiography of the affected limb is needed to look for any complications from the disease. As seen with our case the patient was also having osteomyelitis of the proximal left femur which had to be treated as well. We proceeded with an ultrasound of the affected limbs for reasons of economy and easy availability. Typical findings on ultrasound would include bulky muscle with hypoechoic focal lesions and internal debris. Gordon *et al* demonstrated that computed tomography (CT) or magnetic resonance imaging (MRI) are the imaging of choice for early diagnosis. CT provides better delineation of muscle than plain radiographs and would be useful in differentiating between tumours, hematoma, thrombophlebitis or abscess. However, it may fail to demonstrate early inflammatory changes^2^. MRI would be a better modality than CT as there was a case reported in which areas of signal attenuation were visible on MRI but not on CT scan^1^. Thus, MRI is the imaging of choice as it is able to show the extent of involvement, site of collection, and help differentiate other pathological conditions from pyomyositis. Imaging studies are not pertinent to establish the diagnosis but can be used as a useful adjunct. The gold standard is still aspiration of pus from the involved muscle followed by a positive culture.

Once a diagnosis has been established, we move on to the management of the disease. The mainstay of management of the disease is surgical drainage followed by parenteral antibiotics. If a patient presents in the invasive stage, early diagnosis and prompt intravenous antibiotic administration can prevent abscess formation and avoid surgical drainage. Parenteral Cloxacillin is the initial recommended treatment based on the most culpable organism. In regard to our patient, he was first given parenteral Ceftazidime which is a third-generation cephalosporin to cover for broad spectrum of organisms including melioidosis which is an abscess producing organism endemic to this part of Malaysia. The antibiotics were changed subsequently to Cloxacillin in line with literature once the intra-operative cultures were obtained. He also had multiple abscesses which was drainable thus was sent for incision and drainage of the abscesses as well. Cultures were taken intra-operatively to guide antibiotic treatment. There is literature evidence that antibiotic treatment should be continued until there is complete reduction of abscess, normal leukocyte values and the patient being afebrile for at least one week. However, if a patient presents in the late phase as seen with our case, the recommendation is four to six weeks of parenteral antibiotics. In regard to the case reported above, he only fulfilled two out of three criteria reported by previous reports. His white blood cell count was going down in trend and there was complete reduction of abscess however he was still having multiple temperature spikes in ward despite being on antibiotics for close to six weeks. At that time, we were ruling out any hospital acquired infection such as pneumonia, urinary tract infection or infected sores that might explain his unresolving fever however examination and investigations sent pointed to none of that. When we found out that he had tuberculosis and initiated ATT, he no longer was febrile. He was allowed discharge a few days later.

The pertinent take home message from this case report would be, pyomyositis is a rare infection of the skeletal muscle that is difficult to diagnose as it mimics a wide array of conditions and presents with vague signs and symptoms. It must always be kept in the back of a clinician’s mind whenever a patient presents with muscle pain especially in the immunocompromised population. The nature of the disease calls for quick diagnosis and initiation of treatment due to the possibility of septicaemia if left undiagnosed. The prognosis of the disease is excellent if treated early.
